# Deciphering the Tumor–Immune–Microbe Interactions in HPV-Negative Head and Neck Cancer

**DOI:** 10.3390/genes14081599

**Published:** 2023-08-08

**Authors:** Min Hu, Samuel Coleman, Muhammad Zaki Hidayatullah Fadlullah, Daniel Spakowicz, Christine H. Chung, Aik Choon Tan

**Affiliations:** 1 Department of Oncological Sciences, Huntsman Cancer Institute, University of Utah, Salt Lake City, UT 84112, USA; min.hu@hci.utah.edu (M.H.); sam.coleman@hci.utah.edu (S.C.); zaki.wilmot@hci.utah.edu (M.Z.H.F.); 2 Department of Biomedical Informatics, Huntsman Cancer Institute, University of Utah, Salt Lake City, UT 84112, USA; 3 Pelotonia Institute for Immuno-Oncology and Division of Medical Oncology, The Ohio State University Comprehensive Cancer Center, Columbus, OH 43210, USA; daniel.spakowicz@osumc.edu; 4 Department of Head and Neck Endocrine Oncology, Moffitt Cancer Center, Tampa, FL 33612, USA; christine.chung@moffitt.org

**Keywords:** head and neck cancer, tumor-associated microbes, tumor microenvironment

## Abstract

Patients with human papillomavirus-negative head and neck squamous cell carcinoma (HPV-negative HNSCC) have worse outcomes than HPV-positive HNSCC. In our study, we used a published dataset and investigated the microbes enriched in molecularly classified tumor groups. We showed that microbial signatures could distinguish Hypoxia/Immune phenotypes similar to the gene expression signatures. Furthermore, we identified three highly-correlated microbes with immune processes that are crucial for immunotherapy response. The survival of patients in a molecularly heterogenous group shows significant differences based on the co-abundance of the three microbes. Overall, we present evidence that tumor-associated microbiota are critical components of the tumor ecosystem that may impact tumor microenvironment and immunotherapy response. The results of our study warrant future investigation to experimentally validate the conclusions, which have significant impacts on clinical decision-making, such as treatment selection.

## 1. Introduction

Head and neck squamous cell carcinoma (HNSCC) includes malignancies in the oral cavity, pharynx, and larynx [1]. In 2023, HNSCC is projected to account for over 11,500 deaths in the United States alone [2]. Over the past few decades, risk factors for HNSCC have been intensely studied. While smoking or alcohol consumption strongly correlates with HNSCC, studies have presented compelling evidence that Human Papilloma Virus (HPV) infection plays an important role in tumorigenesis [3,4,5]. However, HPV positivity is typically determined by whether HPV-DNA is detected, and some studies argue that this method alone leads to false positives, especially in non-cervical cancers [6,7,8]. In HNSCC, with more refined detection methods, HPV-positive cases are largely confined to only a subset of HNSCC—oropharyngeal cancer [7], particularly of the base of the tongue and of the tonsils [3,9]. Thus, considering HPV-negative cases are the major subtype that contributes to HNSCC mortalities [10], and FDA-approved HPV vaccines are highly effective in preventing HPV infection, a better biological understanding of HPV-negative HNSCC is urgently needed.

Cancer cell-intrinsic factors such as genetic mutation have traditionally been the focus of cancer research [11]; however, the field of tumor microenvironment (TME) has advanced dramatically in recent years [12,13]. One salient example is the understanding of the immune system concerning cancer, which has led to a transformation in cancer therapeutics [14,15,16,17]. Currently, in addition to the traditional modalities, such as surgery, radiation, or chemotherapy, patients with unresectable or metastatic HNSCC can be treated by the FDA-approved immune checkpoint inhibitors pembrolizumab and nivolumab [18,19,20,21]. However, despite the success of immunotherapy, many patients’ tumors do not respond, or they eventually develop resistance. Thus, elucidating resistance mechanisms has been a priority for improving patient outcomes.

HNSCC is a heterogeneous disease, and many factors can impact tumorigenesis, progression, and treatment response [1]. In the TME, aside from cell populations, physical conditions, such as oxygen availability, have emerged to be critical determinants of the state of the tumor [22,23,24,25,26]. Due to the size of tumors and abnormal vasculature in the surroundings [27], hypoxia is a common selective pressure in cancer. Tumors that can adapt to a hypoxic environment are associated with poor clinical prognoses [28,29]. Molecularly, hypoxia is associated with genomic instability and increased metastatic potential [30,31]. Importantly, hypoxia has also been shown to promote immunosuppression, which worsens immunotherapy response and contributes to resistance [32].

In a study that investigated hypoxia across 19 cancer types, HNSCC ranked the highest based on hypoxia score [22]. Considering the immunosuppressive characteristic of hypoxia, understanding how hypoxia affects immunotherapy can impact patient outcomes profoundly. Consequently, Brooks et al. developed and validated a 54-gene transcriptional classifier to identify HNSCC tumors with hypoxia or immune phenotypes [33]. Using a modified version of the same classifier, Chaudhary et al. thoroughly examined the molecular features of the Hypoxia phenotype, and they found a multitude of changes in the TME induced by hypoxia, including decreased numbers of pro-inflammatory immune cell types and increased activity of the immunosuppressive cell types [34]. Overall, these results suggest that tumors in the Hypoxia group would not respond to immune checkpoint inhibitors, and this was confirmed in the study by retrospective analyses of clinical outcomes of the tumors treated with pembrolizumab or nivolumab [34]. Brooks et al. and Chaudhary et al. provided substantial evidence for the role of hypoxia in treatment selection [33,34]. The Immune group categorized by the transcriptional classifier is generally more responsive to immunotherapy. While the Hypoxia group has an unideal TME for immunotherapy, EGFR signaling has been identified to be a potential therapeutic target that could remodel the TME to be pro-inflammatory [34]. However, in addition to Hypoxia and Immune groups, there is an intermediate group—Mixture, that is less characterized compared to the other two groups. Thus, it remains elusive how to optimize treatment selection for tumors that belong to the Mixture group.

In the TME, another component—the human microbiota—has attracted increasing attention. With the advent of omics technologies that allow for better detection, the field has experienced a drastic expansion [35,36,37,38,39]. Tumor-associated microbiota have been shown to have a wide variety of effects on both the tumor and the microenvironment [35]. Because of the location of HNSCC, this area of research is especially relevant, as exemplified by HPV. Indeed, many studies have pointed out the diverse roles that the microbiota plays in HNSCC [40,41,42,43,44,45,46]. In this study, we aimed to decipher the intricate interplay between the immune system, tumor, and microbiota in HPV-negative HNSCC. We first identified tumor-associated microbes that are uniquely enriched in the HNSCC tumors in the Immune and Hypoxia groups. Next, we thoroughly examined how these microbes affect the tumor and the TME. Surprisingly, we discovered a signature of three microbes that can robustly stratify the Mixture group based on the state of the TME. Our results provide evidence that microbial composition can aid in making clinical decisions, such as treatment selection. Furthermore, the microbial signatures we discovered in this study could also potentially facilitate the development of synthetic microbial peptides for cancer vaccines [47,48,49,50,51].

## 2. Materials and Methods

### 2.1. Data Sources

#### 2.1.1. Gene Expression

Normalized RNA-seq data for The Cancer Genome Atlas (TCGA) HNSCC cohort were publicly available and downloaded from cBioPortal (accessed on 1 May 2023. https://cbioportal-datahub.s3.amazonaws.com/HNSCC_tcga_pan_can_atlas_2018.tar.gz). We obtained a total of 422 HPV-negative samples with microbe data, 17 of which do not have clinical data. Patients whose samples are included in this study are characterized in Supplementary Table S1.

#### 2.1.2. Microbe Abundance

We obtained 422 pre-processed and decontaminated HPV-negative HNSCC microbiome data from the Poore et al. study [38]. In that study, the authors first extracted both DNA and RNA sequences that were not aligned to the human. Then, these sequences were aligned to microbes using Kraken [52], resolved at the genus-level. A total of 1406 microbe species were included. Solid tissue normal, blood derived normal, metastatic, and primary tumors were used in the HNSCC cohort. SourceTracker2 was used for decontamination [53]. Briefly, the TCGA samples were compared to normal body microbiome data from NIH’s HMP2 project [54]. Furthermore, laboratory and environmental contaminants were also accounted for using the database from SourceTracker2. We refer the readers to the original publication for further details about the algorithm. The data is publicly available and can be downloaded from http://ftp.microbio.me/pub/cancer_microbiome_analysis/ (accessed on 1 May 2023).

### 2.2. Molecular Classification of HPV-Negative Cohort

All samples were classified with a previously described approach [34]. Briefly, we used a published hypoxia–immune transcriptional classifier on the TCGA cohort of samples used in the current study [33]. The classifier consists of gene expression signatures that characterize hypoxia or immune phenotypes in head and neck cancers specifically, and these signatures were also shown to have a predictive ability for prognosis [33]. The original classifier includes 54 genes; however, only 49 genes were detected in the cohort used in the present study. Thus, classification was performed using the 49-gene signature. Samples were classified into one of three groups: Hypoxia (*n* = 157), Mixture (*n* = 200), and Immune (*n* = 65).

### 2.3. Microbial Abundance Analysis

To determine microbes that are significantly enriched in each of the three molecularly classified groups, we used the Limma R package [55]. Since the Limma-trend function is a method originally developed for differential gene expression analysis, microbes and their log2 abundances were treated as genes and gene expressions, respectively. *p*-values were adjusted for multiple testing using the Benjamini–Hochberg correction [56]. We retained only microbes with adjusted *p*-values lower than 0.05 and log2 fold changes larger than 0.5. We then categorized these microbes into two unique microbial signatures enriched for the Immune and Hypoxia groups.

### 2.4. Gene Set Enrichment Analysis

To discern pathway enrichment in the three molecular groups, we performed single-sample gene set enrichment analysis (ssGSEA) using the GSVA R package [57]. Gene expression values were first transformed into Z-scores across samples. Then, three collections of gene sets were used for enrichment: Hallmark, TIMEx, and Immune. The Hallmark gene sets were curated to represent a wide variety of biological processes and can thus provide insight into how the three groups differ broadly [58]. The TIMEx gene sets were originally established by deconvoluting bulk RNAseq into cell types using single-cell RNA-seq data signatures [59]. Therefore, the TIMEx gene sets can reflect cell composition changes in the TME between the three groups. Lastly, the Immune gene sets were a list of previously published gene sets that are associated with better responses to immunotherapy across multiple cancer types [60]. Spearman rank correlation coefficients were calculated to determine the associations between ssGSEA results and the abundance of differentially enriched microbes.

### 2.5. Microbial Diversity Analysis

To quantitate microbial diversity and composition, we performed an alpha diversity analysis using Shannon Index. The Shannon Index is a quantitative measure that describes both the abundance of each species and the number of species present in a community. To calculate the Shannon Index for each sample, we used the Vegan R package [61].

### 2.6. Survival Analysis

Survival analyses were performed using overall survival (OS) information of the patients whose samples were used in this study. Samples were divided at the median microbe abundance into high and low groups. We generated Kaplan–Meier plots with the “survival” package in R to visualize survival curves [62], and we performed log-rank tests to test the statistical significance of the difference in survival between the two groups.

## 3. Results

### 3.1. TME Landscape of HPV-Negative HNSCC

**Figure 1:**
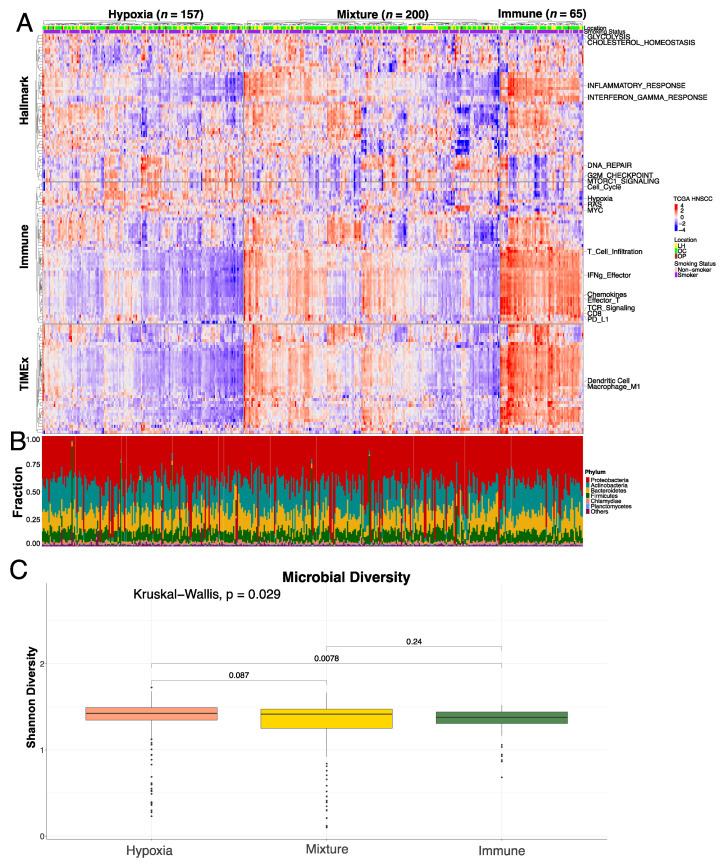
Landscape of tumor–immune–microbe (TCGA HPV-negative cohort) in Hypoxia/Mixture/Immune groups. (**A**) Heatmap showing ssGSEA enrichment in three collections of gene sets. Each cell of the heatmap represents the enrichment score (blue: −4, red: 4) for each sample (*x*-axis) in the corresponding pathway (only representative pathway names are shown on the *y*-axis for visualization purposes; Supplementary Figure S1 is the same heatmap with all pathway names annotated). The top two rows are smoking status and location (LH = Larynx/Hypopharynx, OC = Oral Cavity, OP = Oropharynx) information. (**B**). Stacked bar plot represents the fraction (*y*-axis) of each top 6 phyla as well as collapsed “others” group in each sample (*x*-axis). (**C**) Box plot showing the distribution of each molecular group’s (*x*-axis) Shannon Index (*y*-axis). The result of the Kruskal–Wallis test is shown at the top of the plot. The Hypoxia group shows higher diversity compared to the Immune group based on pairwise tests.

To define the tumor–immune–microbe interactions, we comprehensively characterized the global TME landscape of 422 TCGA HPV-negative HNSCCs. We first molecularly classified HPV-negative HNSCCs into Hypoxia (*n* = 157), Mixture (*n* = 200), and Immune (*n* = 65) groups based on gene expression signatures to reflect the different TME phenotypes [34]. Next, we performed ssGSEA using gene expression data using Hallmark, TIMEx, and Immune gene sets (Figure 1A). As expected, we observed a significant separation of TME phenotypes between the Hypoxia and Immune groups (Figure 1A). Specifically, pathways and gene sets involved in immune processes, such as Immune infiltration, TFG-β signaling, Inflammatory Response, Chemokines, etc., were enriched in the Immune group. Conversely, cell-intrinsic properties, such as Cell cycle, Hypoxia, Glycolysis, and intracellular signaling, were enriched in the Hypoxia group (Figure 1A). The Mixture group shows a “mixture” enrichment of these gene sets and pathways.

In addition to the molecular differences among the three groups, we also sought to characterize how these molecular groups vary in microbial composition, as tumor-associated microbiota have been shown to play important roles in tumor progression. In each molecular group, we determined the top six phyla of microbes (*Actinobacteria*, *Bacteroidetes*, *Chlamydiae*, *Firmicutes*, *Planctomycetes*, and *Proteobacteria*), and we did not observe any differences in the phyla that are most populated in the groups (Figure 1B). We also quantified the diversity of microbes in each group using the Shannon Diversity Index [63], and there was a statistically significant difference between the Hypoxia and Immune group, with Hypoxia having higher diversity (Figure 1C).

### 3.2. Tumor-Associated Microbiome of HPV-Negative HNSCC

**Figure 2:**
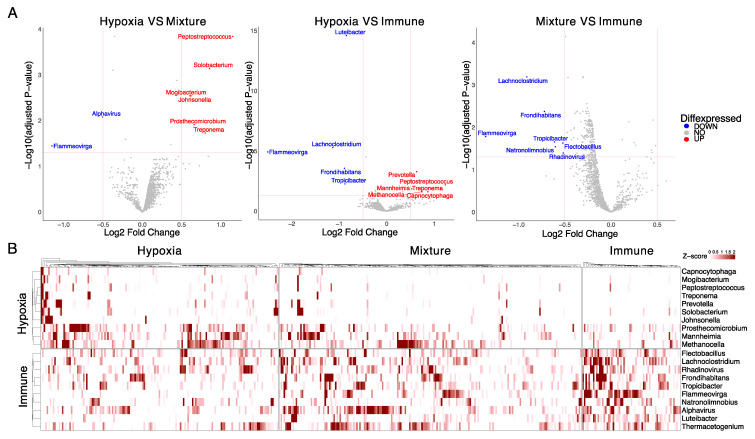
Microbial signature of Hypoxia and Immune groups. (**A**) Volcano plots of Log2 fold changes in pairwise comparisons of all three molecular groups. Vertical and horizontal red lines represent the cutoff threshold of absolute Log2 FC >0.5 and adjusted *p*-value < 0.05 respectively. (**B**) Heatmap representing the abundance of the microbial signatures across the three molecular groups. Each cell of the heatmap is the Z-score of the abundance of the microbe (*y*-axis) in a sample (*x*-axis).

With the result that the diversity at the phylum level is different between the Hypoxia and Immune groups, we wanted to further investigate whether there are microbe species that are differentially enriched in each group. We performed differential abundance analyses on the 1406 microbes and found 20 that can be grouped into Hypoxia-enriched or Immune-enriched categories (Figure 2A). Of the 20 microbes, 10 microbes are uniquely enriched in the Hypoxia group, and the other 10 are increased in the Immune group (adj. *p*-value < 0.05, log2FC > 0.5). The trends of the abundance of these microbes across samples are consistent with the molecular classification of the TME phenotypes of HPV-negative HNSCC (Figure 2B). Moreover, we compared the composition of these microbes in groups classified based on other features of HNSCC, including smoking status and tumor anatomical location. While we found that smoking status does not impact the composition of the 20 microbes, we did observe a difference between oral tumors versus non-oral tumors. However, the results of oral tumors versus non-oral tumors were similar to the study by Kim et al. [40]. Thus, we refer our readers to that study.

### 3.3. Molecular Features Associated with Microbial Signatures

**Figure 3:**
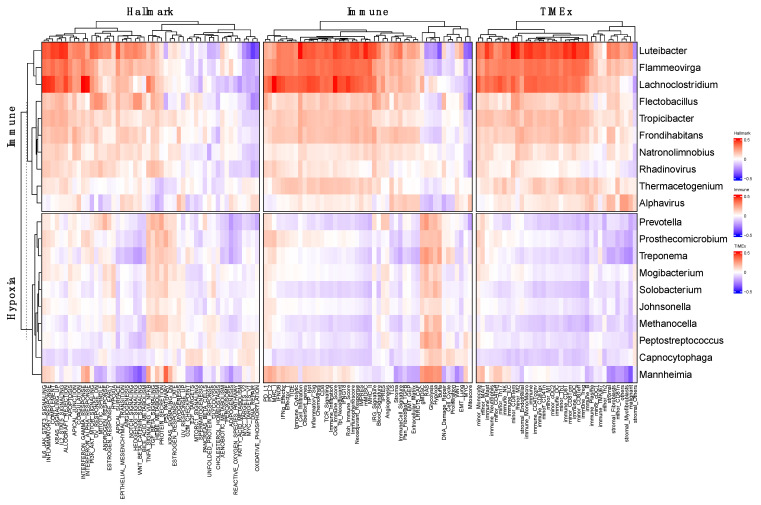
Correlation between microbial signature and Hallmarks, TIMEx, and Immune gene sets. Heatmap showing pathways enrichment of the 20 microbes in the microbial signatures. Each cell is the Spearman Correlation Coefficient (blue = −0.5, red = 0.5) between the microbe (*x*-axis) in the corresponding pathway (*y*-axis).

Since there is a clear divergence between the Hypoxia and Immune groups in their pathway/gene set enrichments, we hypothesized that the microbial signatures should mirror these molecular differences. To test this, we used the same three collections of gene sets (Hallmark, TIMEx, and Immune) and analyzed the correlations between the microbes and the gene sets. We found that these 20 microbes can effectively differentiate the two molecular categories and are highly correlated with pathways and gene sets, including immune-related pathways, Hypoxia, and Glycolysis, according to the Immune and Hypoxia groups (Figure 3). More strikingly, we noticed that the three microbes *Luteibacter*, *Flammeovirgo*, and *Lachnoclostridium* are especially enriched in the Immune group, particularly based on the two immune-focused collections—TIMEx and Immune (Figure 3).

### 3.4. Association of Microbial Signature in TME Remodeling

**Figure 4:**
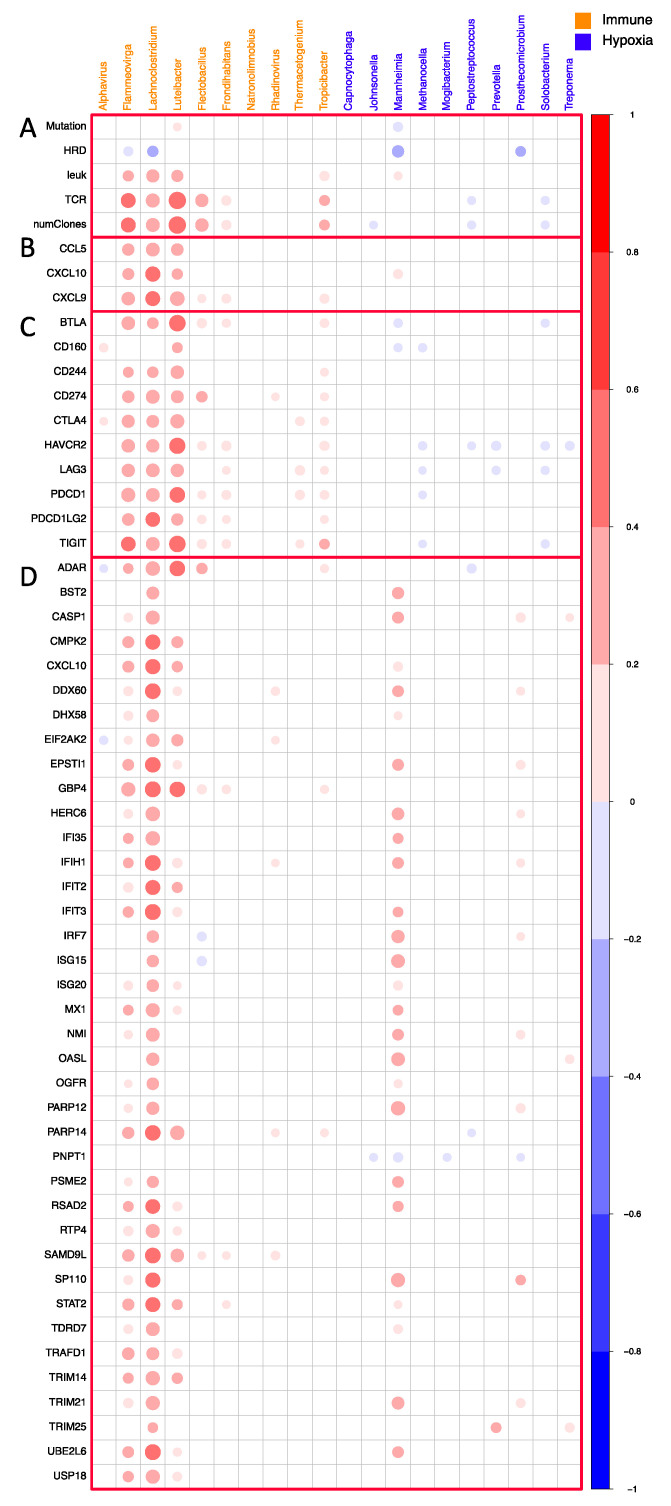
Correlation between microbial signature and TCR repertoire (**A**), T cell recruiting chemokines (**B**), immune checkpoints (**C**), and IFN (**D**). Both the area and the color intensity correspond to the correlation coefficients in all plots of this figure. Correlations with a *p*-value above 0.01 are not shown.

Since the microbial signatures are correlated with various immune-related pathways, the TME is likely remodeled to an inflammatory state. To gain insight into changes in the cell populations in the TME, we analyzed the association between the microbial signature and cell type composition using a previously published dataset [59]. This dataset was generated by applying various tumor deconvolution methods (CIBERSORT, xCELL, TIMER, EPIC, Consensus^TME^, quanTIseq, and MCP counter) to the bulk RNA-seq samples [64,65,66,67,68,69,70]. Here we see the Immune microbial signature is largely positively correlated to immune cell populations, suggesting that higher immune cell infiltration was associated with the Immune microbes (Supplementary Figure S2). As previous studies have indicated that tumors with inflammatory TMEs and high immune cell infiltration are more likely to respond to Immunotherapy [34,71], we further explored the relationship between the enrichment of these microbes and other factors that are essential to immunotherapy response. First, we correlated the 20 microbes with common features associated with immunotherapy responses, including Total Mutation Burden (mutation), homologous recombination deficiency (HRD) score, leukocyte infiltration (leuk), total T cell receptors (TCR) reads, and number of TCR clones. We observed that the three microbes *Luteibacter*, *Flammeovirgo*, and *Lachnoclostridium* enriched in the Immune group are highly correlated with TCR reads, number of clones, and as leukocyte infiltration (Figure 4A). Next, we also found that these three microbes are highly correlated with chemokines for CD8^+^ T-cell infiltration (*CXCL9*, *CXCL10*, and *CCL5*) (Figure 4B). This suggests that the three microbes might play a role in remodeling TME for these samples.

We next investigated the correlation between the 20 microbes with the target of immune checkpoint blockade and found that the same 3 microbes showed high correlations with these immune checkpoints. For example, *Luteibacter* showed a high correlation with *BTLA*, *HAVCR2*, *PDCD1*, and *TIGIT* (Figure 4C). This points to the role of microbes in regulating immune checkpoints in TME. Finally, we performed the correlation between the microbial signatures and interferon (IFN)-stimulating genes (ISG) [72,73]. We found that the same three microbes showed the highest correlations with ISG, especially *Lachnoclostridium* (Figure 4D).

### 3.5. Overall Survival Stratified by Microbial Signature

**Figure 5:**
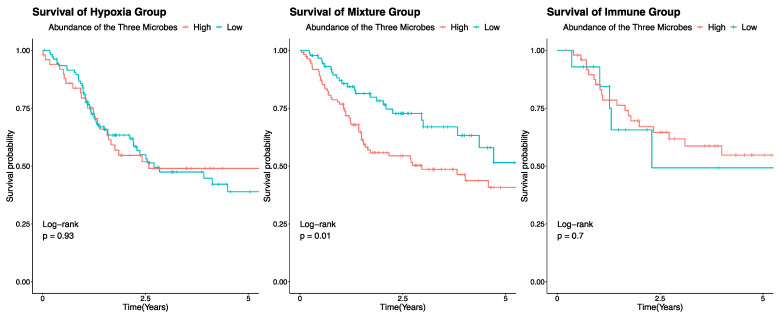
Five-year survival of patients based on the co-abundance of the three microbes. Kaplan–Meier curves of patients in each molecular group. Results of the Log-rank test are shown in each plot.

*Lachnoclostridium, Flammeovirgo*, and *Luteibacter* are significantly enriched in the Immune group compared to the Hypoxia and Mixture groups. With the same three microbes being positively correlated with features indicative of changes in the TME, suggesting that these microbes are the main contributors to the remodeling of the TME, we hypothesized that they could potentially be biomarkers for overall survival. To test the hypothesis, we performed survival analysis using this three-microbial signature in each molecular group (Figure 5). We divided all samples in each group into High and Low groups based on the median abundance of the sum of the three microbes. In both the Immune and Hypoxia groups, we observed no differences in the high and low subsets. However, this is expected as these three microbes are associated with the immune microenvironment. Both Immune and Hypoxia groups are homogenous in terms of their TME states, and no differences within each group showed that the classifier could accurately reflect the immune/hypoxia phenotype. However, in the Mixture group, a comparably more heterogenous group, we observed significant differences in the High and Low groups, with the High group having a worse outcome.

## 4. Discussion

Currently, patients with HPV-negative HNSCCs suffer from worse outcomes compared to those with HPV-positive HNSCCs. Owing to the complex tumor ecosystem in HNSCC, we aimed to decipher the interactions between the immune system, tumor, and tumor-associated microbiota to provide a deeper understanding of HPV-negative HNSCC. Our findings provide compelling evidence that tumor-associated microbiota significantly affect the immune system and cancer prognosis, and it could be used as another biomarker, especially for the less understood subtype of HNSCC.

Cancer is extraordinarily complex because of the multitude of factors that determine the state and behaviors of the tumor ecosystem. Across cancers and particularly in HNSCC, hypoxia has emerged to be a condition that can impact the TME greatly [22]. To facilitate utilizing hypoxia as a biomarker, Brooks et al. created a 54-gene classifier that can distinguish hypoxia or immune phenotypes based on gene expression profiles [33]. Subsequently, Chaudhary et al. investigated the specific immunosuppressive effects of hypoxia and found that EGFR signaling could be potentially exploited to alter the TME to an inflammatory state [34]. In our study, we used the same classifier to categorize TCGA HNSCC HPV-negative samples into Immune, Hypoxia, or Mixture groups. Using ssGSEA with three collections of gene sets—Hallmark, TIMEx, and Immune, we observed a clear divergence between the Immune and Hypoxia groups. More specifically, these two groups greatly differ in their enrichment in immune-related processes, validating the classifier’s ability to distinguish the two phenotypes. Given that the Immune gene sets are pathways associated with a better clinical response to immunotherapy, enrichment in the Immune gene sets strongly corroborates the finding that tumors in the Immune group respond better to immunotherapy from Chaudhary et al. [34].

In addition to tumor cells and immune cells, the tumor-associated microbiota has emerged to be another important component of the tumor ecosystem. Studies have pointed out microbial species that are pro-tumorigenic and others that are anti-tumorigenic [41,44,74,75]. This inspired us to decode the complex relationship between the immune system, tumor, and microbiota. We first characterized the microbial profiles of the three molecular groups (Hypoxia, Mixture, and Immune). We determined the top six common microbe phyla in each group and compared the composition of these phyla. While the most common phyla are the same across all groups, there is a statistically significant difference in the composition between the Hypoxia and Immune groups, with the Hypoxia group having slightly higher diversity. The variations at the phylum level strongly demonstrate the association between tumor-associated microbiota and different TME phenotypes, and this encouraged us to further examine the differences in microbial enrichment at the species level, as many other cancer microbiome studies found differences at lower taxonomy levels [74,76,77,78]. Indeed, we found ten species that are uniquely enriched in the Hypoxia group and ten in the Immune group. These two microbial profiles can clearly separate the three groups by microbial abundance.

Although the abundance of these 20 microbes can classify the Immune and Hypoxia phenotypes, we sought to examine whether these microbes actually correlate with the molecular features associated with the phenotypes and also to gain insight into how these microbes may specifically affect the tumor and the immune system. Using pathway enrichment analysis, we saw a significant association between the abundance of these microbes and immune processes. This observation supports the relationship between the microbiota and the immune system that many other studies have shown. For instance, Wu et al. found that certain colonic commensal bacterium can activate the T helper type17 response, which increases inflammation level and ultimately leads to tumor development [79]. Gur et al. show that a bacterium can bind to an immune checkpoint TIGIT to hide tumors from the detection of the immune cells [80]. In addition to immune processes, pathways representing the Hypoxia phenotypes also show strong signals with positive correlations between the Hypoxia microbial signature and Hypoxia, Glycolysis, and RAS pathways. With low oxygen levels, tumors rely on glycolysis more for ATP generation [26]. In the meantime, Ras is an upstream regulator of the production of HIF1-α [81], and Ras signaling has been shown to play an important role in metabolic robustness during hypoxia [82]. Overall, the associations we observe in this analysis are consistent with the current biological understanding.

T cells are the main effector cells that contribute to the elimination of abnormal cells, such as neoplastic cells [83]. However, in order to first recognize abnormal cells, the receptors on T cells (T cell receptors) need to bind to the antigen presented on the antigen-presenting cells, which will then activate the T cells [84]. However, cancer has evolved to have the ability to engage immune checkpoints on T cells to suppress their activities [16]. In addition, T cell activation requires physical binding to the antigen, which means that the amount of T cells present in the proximity of the tumor is another factor that dictates this process [85]. Thus, another immune-escaping mechanism cancer utilizes is by creating a hostile environment, such as a hypoxia condition, that prevents T-cell infiltration [34]. Given that our microbial signatures are highly associated with Immune pathways, such as PD-L1 and T-Cell infiltration, which are predictive of better responses to immunotherapy, we further explored whether the microbial signatures are associated with genes that are predictive of immunotherapy response. We included a comprehensive panel of genes that included chemokines, interferon gene signature, and immune checkpoints, as well as TCR reads, number of TCR clones, and leukocytes. Strikingly, we observed three microbes— *Lachnoclostridium*, *Flammeovirga,* and *Luteibacter*—that are highly enriched across the board, whereas other microbes show weaker correlations.

Of the three microbes, *Lachnoclostridium* (*Lachnospiraceae*) has already been linked to immunotherapy response. In a clinical trial, researchers performed fecal microbiota transplantation from anti-PD-1 responders to non-responders. This intervention showed clinical benefits in some patients, and the abundance of *Lachnoclostridium* was enriched in these responders. Molecularly, the researchers found higher activated CD8+ T cells in the tumors, suggesting heightened immunity potentially as a result of the microbiome changes [86]. Another clinical trial with a similar design also observed the same changes in the microbiota and immunity in responders [87]. Although both of these two studies only showed a correlation between *Lachnoclostridium* in the gut microbiome and immunotherapy response, it is possible that *Lachnoclostridium* could travel from the gut to the tumor, as many studies have indicated that microbes are able to migrate through the gastrointestinal tract or hematogenously [79,88,89,90]. It would be interesting for future studies to investigate whether changes in the gut microbiome could alter the tumor-associated microbiome. Mechanistically, *Lachnoclostridium* has been shown to increase chemokine production by colorectal cells [91]. Chemokines are proteins that attract T cells, which results in higher T cell infiltration and better response to immunotherapy [92]. Thus, this study provides support for the causal and pro-inflammatory role of *Lachnoclostridium* in modulating the immune environment. Interestingly, in colorectal cancer, *Lachnoclostridium* has been shown to be associated with low tumor burden in a colitis-associated tumor mouse model, and the authors concluded that *Lachnoclostridium* has an anti-inflammatory function due to butyrate production [93]. These studies highlight the complex nature of the tumor ecosystem and the potentially dynamic nature of tumor-associated microbes.

In contrast to *Lachnoclostridium*, the literature on *Flammeovirga* and *Luteibacter* in cancer is sparse [35,37,94]. One study by Zhu and colleagues found that *Flammeovirga,* together with *Lachnoclostridium*, are associated with infiltrating CD8+ T cells [75]. In that study, the authors used a cutaneous melanoma cohort from TCGA and found that *Lachnoclostridium* and *Flammeovirga* are not only positively associated with infiltrating CD8+ T cells but also chemokines *CXCL9*, *CXCL10,* and *CCL5*, which is consistent with our results using the HPV-negative HNSCC cohort. In addition, we also observed that the three microbes are positively correlated with infiltrating leukocytes, T cell receptors, and the number of T cell receptor clones. On the contrary, we did not see any correlation between the microbes and total mutation burden or homologous recombination deficiency, as expected.

Considering that the state of the immune system is significantly associated with survival outcome [34], we tested whether survival probability is associated with the total abundance of the three microbes. As expected, in the more homogenous Hypoxia or Immune groups, we did not observe significant differences between patients with high and low abundance of the microbes. Intriguingly, in the heterogenous Mixture group, patients with a higher co-abundance of the three microbes had worse outcomes. Although it seems counterintuitive, it is critical to note that this TCGA cohort never received immunotherapy. Inflammation in cancer can be a double-edged sword. Chronic inflammation contributes to tumorigenesis and tumor progression, while acute inflammation facilitates response to therapies [71,95,96,97,98,99,100,101,102,103,104,105]. Thus, the high abundance of these three microbes could induce chronic inflammation, which would explain the worse outcomes for these patients. However, we speculate that these patients would benefit greatly from immunotherapy. Future studies are warranted to develop more refined methods to better distinguish pro-tumorigenic and anti-tumorigenic inflammation.

One limitation of the present study is that the bulk RNA-seq dataset does not contain spatial information. Because of this limitation, we intentionally addressed the microbes only by “tumor-associated” instead of “intratumoral”. Additionally, the location of the microbes is also informative, as studies have used this information to gain insight into the spatial organization of the tumor ecosystem [39]. Thus, future studies should incorporate spatial data to more comprehensively understand the complex relationship between the immune system, tumor, and microbiota. Another limitation of the study is potential contamination of the samples. Although a state-of-the-art decontamination method was applied in the study that generated the dataset [53], the method is not perfect. This could potentially explain the sparsity of existing literature linking *Flammeovirga* and *Luteibacter* to cancer. However, this does not invalidate our findings. Like all other conclusions in the study, we encourage experimental researchers to test whether these microbes are involved in cancer.

## 5. Conclusions

The findings from our study introduce the possibility that microbes increase chemokine in the TME, which attracts T cells and increases T cell infiltration. These microbes may also create a more inflammatory microenvironment that promotes the activities of the T cells. Ultimately, patients with higher amounts of these microbes have TMEs that are more poised to respond better to immunotherapy. Though speculative, as our results only show association and not necessarily causation, the present study provides evidence for a complex tumor ecosystem of cancer cells, immune cells, and microbes. Our results encourage future experimental studies to further mechanistically dissect the causal relationships between these components in the tumor ecosystem. In the meantime, it is evident that microbial information could be utilized to stratify a heterogenous and less-understood patient population, the Mixture group. Clinically, patients in this group can potentially incorporate microbial information to direct their treatment selection, similar to the Hypoxia and Immune groups. Lastly, our framework applies not only to HNSCCs, but potentially many other types of cancers, especially those without known microbial drivers. Thus, we invite other researchers to incorporate the framework in their fields of cancer studies.

## Data Availability

Publicly available datasets were analyzed in this study. The data can be found here: https://cbioportal-datahub.s3.amazonaws.com/HNSCC_tcga_pan_can_atlas_2018.tar.gz; http://Ftp.Microbio.Me/Pub/Cancer_Microbiome_Analysis/ (accessed on 1 May 2023).

## Supplementary Materials

The following supporting information can be downloaded at: https://www.mdpi.com/article/10.3390/genes14081599/s1, Supplementary Table S1: Characteristics of the patients whose samples are included in the study. A breakdown of number of samples in each molecular types (Hypoxia, Mixture, and Immune) based on different characteristics. Fisher’s Exact test is performed between molecular types and characteristics to show associations. Supplementary Figure S1: Heatmap showing ssGSEA enrichment in three collections of gene sets. Each cell of the heatmap represents the enrichment score (blue: −4, red: 4) for each sample (*x*-axis) in the corresponding pathway (*y*-axis). The top two rows are smoking status and location (LH = Larynx/Hypopharynx, OC = Oral Cavity, OP = Oropharynx) information. Supplementary Figure S2: Correlation between microbial signature and cell type composition. Heatmap showing cell type enrichment of the 20 microbes in the microbial signatures. Each cell of the heatmap is the Spearmen Correlation Coefficient (blue = −0.5, red = 0.5) between the microbe (*y*-axis) in the corresponding cell type (*x*-axis).
