# Supplementary material for: Deciphering the Tumor–Immune–Microbe Interactions in HPV-Negative Head and Neck Cancer
Source: Genes (Basel). 2023 Aug 8;14(8):1599. doi: 10.3390/genes14081599 (PMC10454300; doi:10.3390/genes14081599)
Supplement: Supplementary file 1 [file genes-14-01599-s001.zip › Supplementary Figures and Table.pdf]

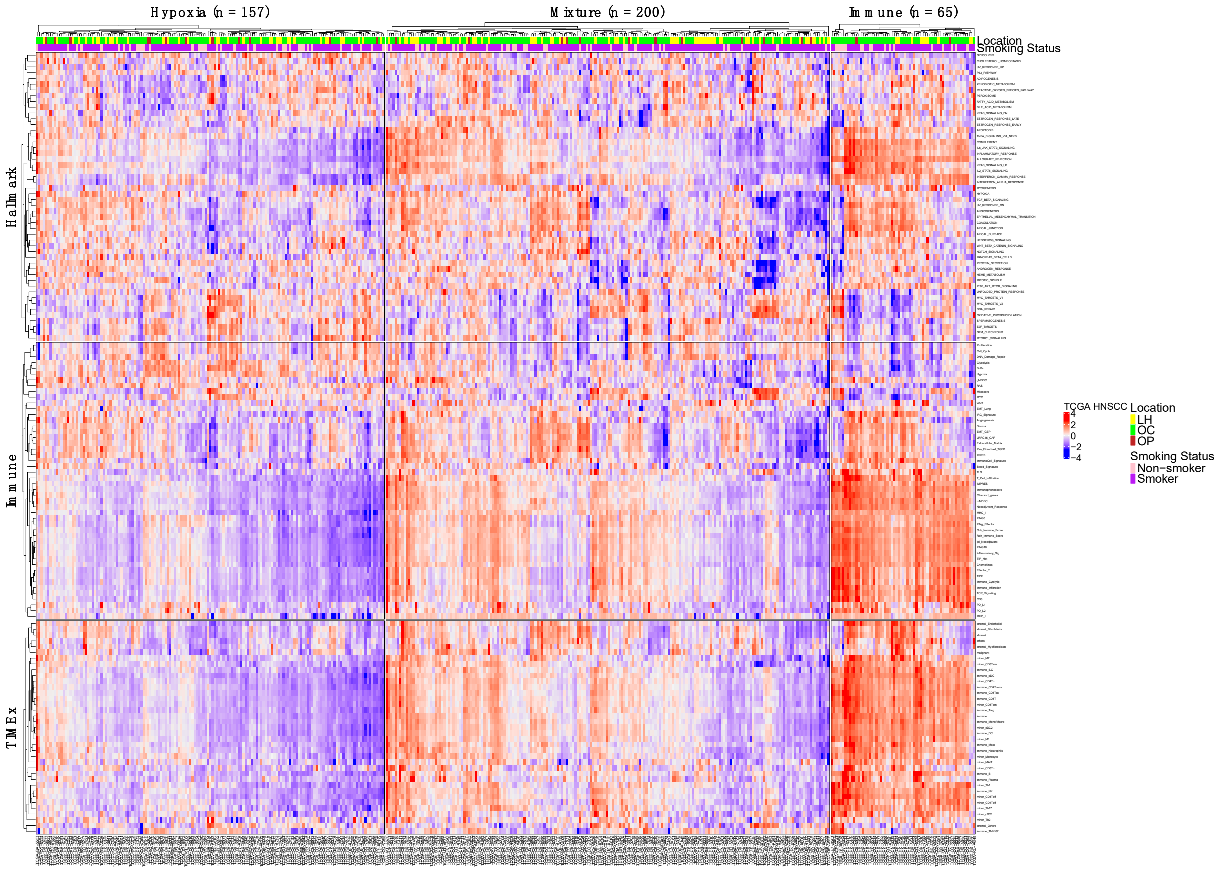

Supplementary Figure S1. Heatmap showing ssGSEA enrichment in three collections of gene sets. Each cell of the heatmap represents the enrichment score (blue: −4, red: 4) for each sample (*x*-axis) in the corresponding pathway (*y*-axis). The top two rows are smoking status and location (LH = Larynx/Hypopharynx, OC = Oral Cavity, OP = Oropharynx) information.

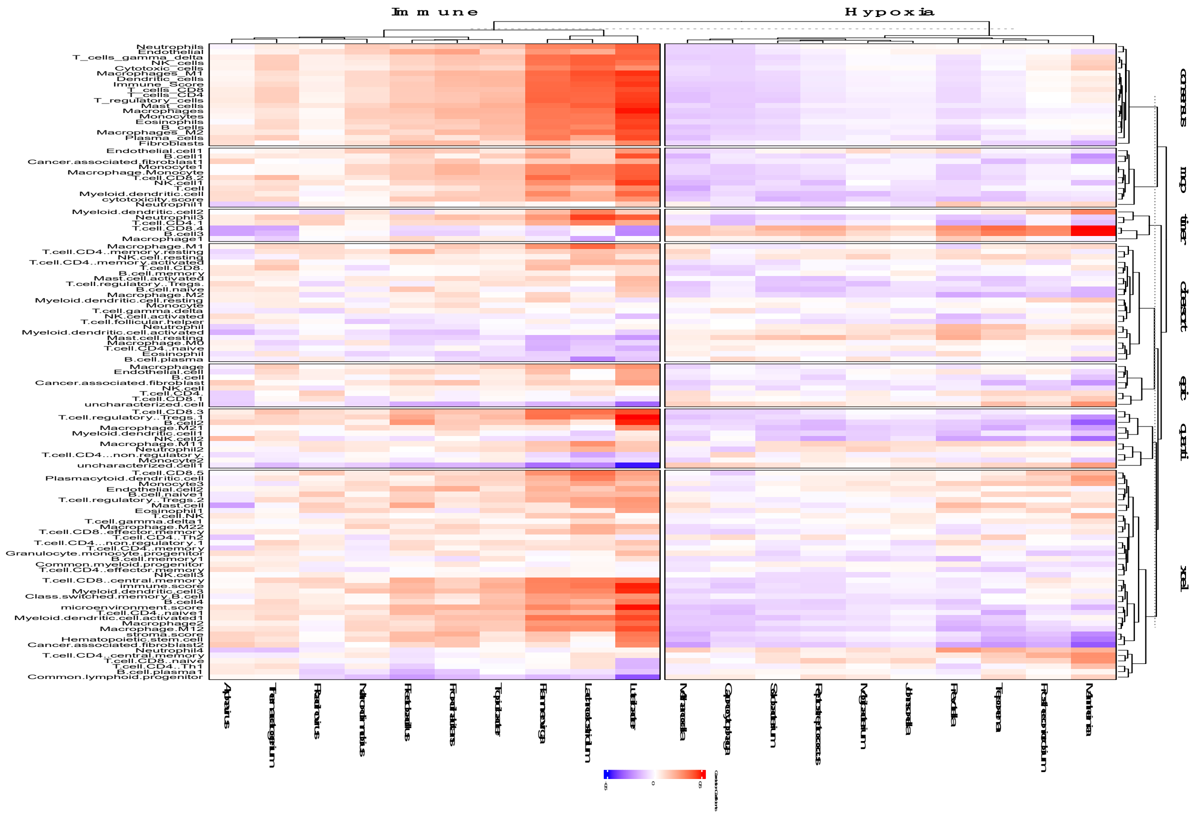

Supplementary Figure S2. Correlation between microbial signature and cell type composition. Heatmap showing cell type enrichment of the 20 microbes in the microbial signatures. Each cell of the heatmap is the Spearmen Correlation Coefficient (blue = −0.5, red = 0.5) between the microbe (*y*-axis) in the corresponding cell type (*x*-axis).

Supplemental Table 1. TCGA Cohort

	Hypoxia/Immune Status				
	Hypoxia	Mixture	Immune	Total	p-value*
Not Available	5	8	4	17	
Age (median, range)	60 (19-87)	62 (29-87)	65 (24-90)	61 (19-90)	
Sex					
Male	112	138	36	286	0.09699
Female	40	54	25	119	
Disease Site					
Oral Cavity	102	118	49	269	
Larynx/Hypopharynx	38	63	10	111	0.05892
Oropharynx	12	11	2	25	
Disease Stage					
I	9	9	5	23	
II	14	30	14	58	
III	31	28	8	67	0.1173
IV	88	101	29	218	
Not Available	10	24	5	39	
Smoking History					
Never smoker	36	36	16	88	0.3393
Ever smoker	116	156	45	317	
Total	157	200	65	422	

*Fisher's Exact test

Supplementary Table 1. Characteristics of the patients whose samples are included in the study. A breakdown of number of samples in each molecular types (Hypoxia, Mixture, and Immune) based on different characteristics. Fisher's Exact test is performed between molecular types and characteristics to show associations.
